# Resting States and Memory Consolidation: A Preregistered Replication and Meta-Analysis

**DOI:** 10.1038/s41598-019-56033-6

**Published:** 2019-12-18

**Authors:** Graelyn B. Humiston, Matthew A. Tucker, Theodore Summer, Erin J. Wamsley

**Affiliations:** 1 Department of Psychology and Program in Neuroscience, Furman University, Greenville, South Carolina United States; 2 Department of Biomedical Sciences, University of South Carolina School of Medicine, Greenville, South Carolina United States

**Keywords:** Wakefulness, Consolidation, Long-term memory

## Abstract

While several recent studies have found that a post-encoding period of quiet, eyes-closed waking rest benefits memory consolidation, others have reported null effects. To more precisely estimate this effect, we conducted a quasi-exact behavioural replication of a recent study from our lab, which found that post-training eyes-closed waking rest improved declarative memory relative to a distractor task. Contrary to our hypothesis, the observed effect was not significant; however, it did fall within the 95% confidence interval of our previous finding. Furthermore, a meta-analytic effect summarizing n = 10 similar studies indicates a moderately sized and significant benefit of waking rest for verbal memory (d = 0.38, p < 0.001). We argue that the apparently conflicting results in this literature are most parsimoniously explained by variability due to sampling and/or measurement error, in a group of studies often underpowered to detect a smaller-than-expected effect of rest. Additionally, exploratory analyses revealed that increased trait daydreaming frequency negatively correlated with memory retention during eyes-closed rest. Together with our replication and meta-analysis, these studies suggest that waking rest confers a small but significant benefit on memory consolidation, and that this benefit requires the mind to be free from attention to either external tasks or spontaneous thought.

## Introduction

Following learning, newly formed memory traces undergo a period of “consolidation”, during which memory is strengthened, stabilized against interference, and in some cases, reorganized in the brain. A nascent body of research suggests that resting with one’s eyes closed following learning may improve memory consolidation, relative to an equivalent period of active wake^1–12^. In several studies using declarative^1,3,4,9,12^, spatial^8^, or motor memory^2^ tasks, participants perform better on a delayed test when learning is followed by a short period of waking rest, rather than by an active wake control condition. This benefit may arise from reduced demand on hippocampal and other memory resources while participants are in an “offline” eyes-closed state of rest, providing an opportunity to consolidate newly learned information^7^. This offline resting state is distinct from active, “online” wake in that participants are not explicitly attending to any external stimuli^1,4,13^, nor to any internally focused cognitive task^14,15^, both of which have been shown to impede consolidation. Of note, disengagement from the external environment and the absence of directed cognition are also key features of sleep, which has long been shown to benefit memory consolidation^16–22^.

Indeed, offline periods of quiet rest manifest many of the features thought to account for the memory benefits of sleep^5–7,23–25^. First and most obviously, both sleep and waking rest are characterized by reduced sensory input, thereby decreasing encoding demands on the hippocampus, which has been proposed as a prerequisite for declarative memory consolidation to take place^7^. Albeit to a lesser extent, like sleep, waking rest is also characterized by slower and more synchronous EEG rhythms relative to active wake, with increasing amounts of alpha (8–12 Hz) and theta (4–7 Hz) predominating over the faster beta (>13 Hz) rhythms of active wake. Neurobiologically, sleep and waking rest are also both marked by decreased acetylcholine levels relative to active wakefulness, and by the presence of hippocampal sharp-wave ripple complexes, both of which are proposed to facilitate memory consolidation^5,6,23–27^. Thus while sleep is a neurobiologically distinct state, key features thought to account for sleep’s memory benefit are also present during quiet waking rest. These features are absent from the typical active wake controls in studies examining the effect of sleep on memory, in which participants may watch videos^22,28^, listen to music^29,30^, or even leave the laboratory^31–34^.

However, waking rest has not consistently been found to benefit memory, with some recent investigations reporting null effects. For example, in one study, post-training waking rest improved memory for a word list after a delay of seven days, but unexpectedly, this occurred only when an immediate memory test was conducted at the end of the first (training) session^13^ (a feature not present in the above-cited studies^1–4,8,9^). In two other studies, waking rest did not lead to a significant improvement over the control condition for the details of a text story, word-picture pairings, or aurally presented words^35,36^. Our own laboratory conducted a study manipulating cognitive load and sensory stimulation during a 15 min period of post-training waking rest, and we found that memory was not improved by periods of low cognitive load or reduced sensory stimulation^37^. These non-significant effects may simply be due to sampling or measurement error, which would be expected to lead to some non-significant findings, even when testing a true effect. They might also be impacted by the relatively low sample sizes across the studies, which have lower power to detect real effects.

To more accurately estimate the effect of waking rest on memory in the current study, we conducted a quasi-exact replication of our 2016 paper (Brokaw *et al*.^1^) that reported a benefit of waking rest for verbal declarative memory^1^, using the same short story task as Dewar *et al*.^4^. Our 2016 study found that 15 min of eyes-closed rest following learning led to better memory for the short story than 15 min spent completing a distractor task^1^. This behavioural observation was the same as that reported by Dewar *et al*. in their 2012 paper^4^. Analysis of EEG recordings across the 15 min rest period in Brokaw *et al*.^1^ also found that improved memory in the waking rest condition was associated with an increased slow EEG oscillation rhythm (0.3–1 Hz) and decreased alpha rhythm (8–12 Hz). Improved memory in the distractor task condition also correlated with more time spent thinking about the past and the future, and less time thinking about the current task. Together, these results suggested that waking states characterized by EEG slowing and spontaneous, unstructured thought facilitate memory consolidation.

The primary aim of this replication was to test whether 15 min of waking rest benefits memory for a short story to a greater extent than 15 min spent completing the distractor task, as found in Brokaw *et al*.^1^. We conducted a preregistered quasi-exact behavioural replication, repeating the procedure of Brokaw *et al*. without conducting EEG recordings, allowing us to test the effect of waking rest on memory performance in a less time- and resource-intensive manner. As in Brokaw el al.^1^, participants listened to a short story, then were given a free recall test prior to engaging in 15 min of either eyes-closed waking rest or a computerized distractor task. Immediately after the end of the experimental manipulation, participants completed a delayed free recall test, and then answered an exit questionnaire about their thought processes during the interval. All participants completed both conditions in counterbalanced order, on separate laboratory visits at least 24hrs apart.

Through the Open Science Framework (OSF), we preregistered the hypothesis, procedure, and analyses for this project in advance of data collection (https://osf.io/w7x4p/), all of which closely followed the procedures of Brokaw *et al*. (2016). We predicted that, as in Brokaw *et al*.^1^, resting after hearing a short story would lead to significantly better free recall of story details at the delayed test. We also predicted that improved memory in the distractor task condition would correlate with more time spent thinking about the past and the future, and less time thinking about the current task.

We also anticipated spontaneous thought as a potential mediator of the effect of waking rest on memory, as spontaneous thought may arise from the reactivation of newly encoded memories^38,39^, and because Brokaw *et al*.^1^ found that more time spent mindwandering during the distractor task predicted better memory at the delayed test. We included questionnaires assessing trait propensity for mindwandering (Mindfulness Attention Awareness Scale (MAAS); a 15-item scale designed to assess mindfulness, a concept which is inversely correlated with “mind wandering”)^40^ and daydreaming (Daydreaming Subscale of the Imaginal Processes Inventory (DFS); assessing trait daydreaming frequency)^41^, which allowed us to conduct exploratory analyses of the relationship between spontaneous thought and the memory benefits of rest. We predicted that greater daydream frequency and mindwandering propensity would positively correlate with memory for the short story.

## Results

### Analysis

All of the primary confirmatory analyses were preregistered (https://osf.io/w7x4p/). Unplanned analyses are noted.

Short story free recall responses were scored by two raters blind to condition, who counted the number of correctly and falsely recalled items. Each short story contained 25 discrete pieces of information. To score correct recall, one point was given for each correctly recalled item, with total possible scores ranging from 0 to 25 points, following the procedure in the Wechsler Memory Scale Manual^42^. To score false recall, one point was given for each time a participant mentioned one of the 25 story elements, but in an incorrect manner (such as recalling the name “Robin” instead of “Robert”). Two raters scored the responses for correct recall (interrater agreement 97%), and one of these raters and another new rater scored the responses for false recall (interrater agreement 73%). Final scores used for analysis were the mean of the two raters’ scores.

Three recall scores were calculated for each test: the number of correctly recalled items, the number of falsely recalled items, and the total number of recalled items (correct + false). Raw change in recall was calculated as (delayed test score - immediate test score), and percent change was calculated as ((delayed score - immediate score)/immediate score).

### Preregistered confirmatory analyses

**Figure 1:**
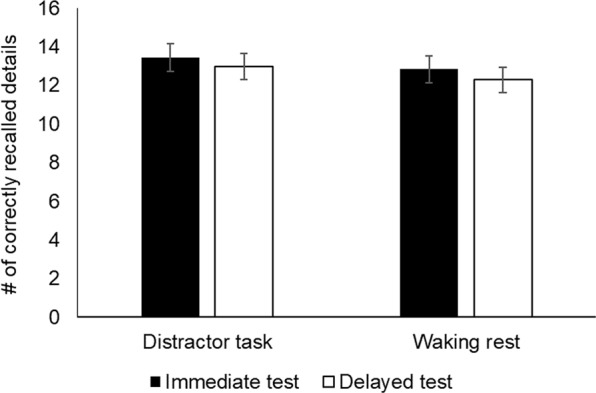
Story Recall. Neither number of correctly recalled items nor memory change across the retention interval differed between the waking rest and distractor task conditions. Error bars ± SEM.

Rest did not benefit raw change in memory relative to the distractor task (correct recall: *t*(30) = 0.37, *p* = 0.71, *d* = 0.09; total recall: *t*(30) = 0.07, *p* = 0.95, *d* = 0.02) (Table 1, Fig. 1). This was contrary to our hypothesis and to the findings of Brokaw *et al*.^1^. As predicted, there was no significant difference between the waking rest and distractor task conditions at the immediate test (correct recall: *t*(30) = 0.83, *p* = 0.41, *d* = 0.15; total recall: *t*(30) = 1.26, *p* = 0.22, *d* = 0.25).

**Table 1:** Raw change over the retention interval for correct, false, and total recall.

	Waking Rest		Distractor Task		*p*
	*mean*	±*SEM*	*mean*	±*SEM*	
Correct recall	−0.55	0.16	−0.47	0.18	0.71
False recall	−0.02	0.17	−0.08	0.13	0.78
Total recall (Correct + False)	−0.56	0.17	−0.55	0.20	0.95

We conducted additional paired-samples *t*-tests to assess the effect of experimental condition on the % change (rather than raw change, see Methods) in correct, false, and total recall across the retention interval. As predicted, the results mirrored those for raw change: % change in correct, false, and total recall did not differ significantly between the waking rest and distractor task conditions. There was also no difference between conditions in baseline false recall or raw change in false recall (Supplementary Table S1).

As predicted, participants in the waking rest and distractor task conditions did not differ in sleepiness (SSS) at the immediate test (*t*(30) = 0.47, *p* = 0.64, *d* = 0.11), but did differ at the delayed test (*t*(30) = 2.04, *p* = 0.05, *d* = 0.40), when participants in the waking rest condition reported being sleepier. This was substantiated by a non-preregistered 2 (immediate test vs. delayed test) x 2 (waking rest vs. distractor task) ANOVA revealing a Time x Condition interaction: *F*(30) = 5.87, *p* = 0.02, *ƞ*_*p*_^2^ = 0.16. However, as expected, there was no significant correlation between sleepiness at test and raw change in correctly recalled items across the retention interval, *r*(31) = 0.10, *p* = 0.56.

Contrary to our hypothesis, there was no correlation between the time spent thinking about Snood, as reported in the distractor condition exit questionnaire, and the raw change in correct recall in the distractor condition, *r*(31) = −0.07, *p* = 0.70.

### Preregistered exploratory analyses

#### Trait mindwandering and daydreaming frequency predict memory retention over quiet rest

**Figure 2:**
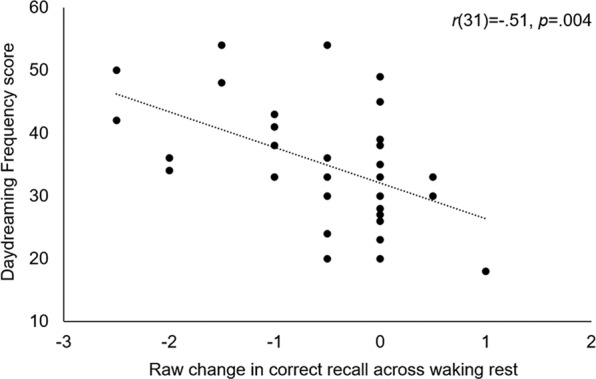
Daydreaming and Story Recall. Trait mindwandering correlated negatively with raw change in correct recall over waking rest.

Trait mindwandering and daydreaming propensity both predicted memory retention across rest. Daydreaming frequency was strongly *negatively* correlated with change in correct recall over waking rest (raw change: *r*(31) = −0.51, *p* = 0.004; % change: *r*(31) = −0.47, *p* = 0.008; Fig. 2), and conversely, was positively correlated with % change in false recall over waking rest, *r*(31) = 0.41, *p* = 0.03. This was contrary to our hypothesis that daydreaming frequency would correlate positively with change in correct recall over waking rest (raw and %), and that it would not correlate with change in false recall. Daydream frequency was also negatively correlated with raw change in total recall in the distractor task condition, *r*(31) = −0.39, *p* = 0.03. To explore the association between trait daydream frequency and subjective experience during the retention interval, we additionally ran non-preregistered correlations between daydream frequency score and subjective experience as assessed via the exit questionnaire. Daydream frequency correlated negatively with time in a “mind blank” state, *r*(31) = −0.42, *p* = 0.02, but was not associated with any other category of experience (Supplementary Table S2).

**Figure 3:**
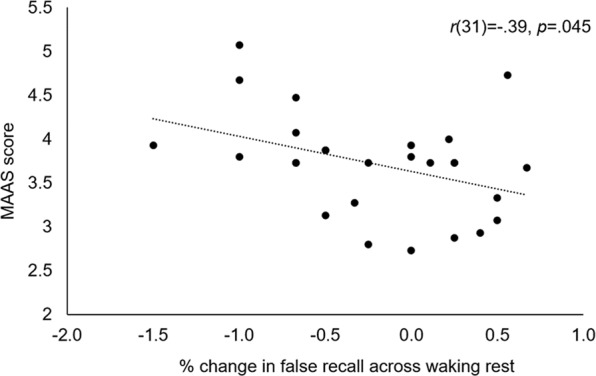
Mindwandering and Story Recall. MAAS score correlated negatively with % change in false recall over waking rest.

Trait mindwandering scores from the MAAS correlated negatively with % change in false recall over waking rest, *r*(31) = −0.39, *p* = 0.045 (Fig. 3). As predicted, trait daydreaming correlated negatively with MAAS score, such that participants with high daydreaming frequency scored low on mindfulness (and thus high on mindwandering propensity) (*r*(31) = −0.48, *p* = 0.006). Additional analyses are reported in Supplementary Table S3.

#### Differences in subjective experience between the rest and distractor conditions

In line with our prediction and the above-reported tendency for participants to be sleepier following the rest vs. distractor condition, participants also rated their ability to concentrate at the delayed test as lower after waking rest, relative to after the distractor (*t*(30) = 2.27, *p* = 0.03, *d* = 0.33). There was, however, no difference between conditions in ratings of how refreshed participants felt (*t*(30) = 0.19, *p* = 0.85).

On the rehearsal questionnaire, participants reported thinking about the story (*t*(30) = 2.99, *p* = 0.006, *d* = 0.62) and imagining the story (*t*(30) = 2.89, *p* = 0.007, *d* = 0.59) more frequently during rest than during the distractor task (Supplementary Table S4). This thought and imagery was described as a mix of spontaneously occurring and intentional (thoughts: 27% spontaneous, 45% intentional; imagery: 39% spontaneous, 36% intentional). Among participants who endorsed experiencing some story-related thought and imagery, the rate of spontaneous vs. intentional rehearsal did not differ between conditions (Fisher’s exact tests comparing the rate of “spontaneous” vs. “intentional” or “both” responses between rest and distractor conditions, all p’s > 0.36).

As expected, participants reported spending more time thinking about the past (*t*(30) = 2.56, *p* = 0.02, *d* = 0.52) and the future (*t*(30) = 3.10, *p* = 0.004. *d* = 0.75) during waking rest than during the distractor task and, specifically, more about something that happened last year or several years ago (*t*(30) = 2.36, *p* = 0.03, *d* = 0.43), the remainder of the day (*t*(30) = 2.48, *p* = 0.02, *d* = 0.61), and tomorrow to next week (*t*(30) = 2.42, *p* = 0.02, *d* = 0.54). Participants also reported spending more time doing “focused meditation” (*t*(30) = 2.27, *p* = 0.03; no participants reported spending any time doing focused meditation during the distractor task) and counting the time (*t*(30) = 2.42, *p* = 0.02, *d* = 0.64) during waking rest. In contrast, participants reported spending more time thinking about their current activity during the distractor task than during waking rest (i.e. thinking about Snood/resting with eyes closed *t*(30) = 6.38, *p* < 0.001, *d* = 1.49). Additional analyses are reported in Supplementary Table S5.

For the most part, subjective experience ratings were unrelated to changes in memory performance across the retention interval (Supplementary Table S6). In one exception, raw change in correct recall over waking rest negatively correlated with the proportion of time participants indicated “I was sleeping” during the rest period on the exit questionnaire, *r*(31) = −0.42, *p* = 0.02. However, this correlation is likely spurious, being driven solely by a single participant who reported spending 60% of the time sleeping. Non-preregistered analyses confirmed that the non-significant effect of waking rest on memory was unaltered when this participant was excluded from analysis (effect of rest vs. distractor task on correct recall: *t*(29) = 0.22, *p* = 0.82; total recall: *t*(29) = 0.22, *p* = 0.83). Additionally, thinking about the story during waking rest or during the distractor task, as reported on the rehearsal questionnaire, was unrelated to memory (Supplementary Table S7).

#### Meta-Analytic Summary Effect

**Figure 4:**
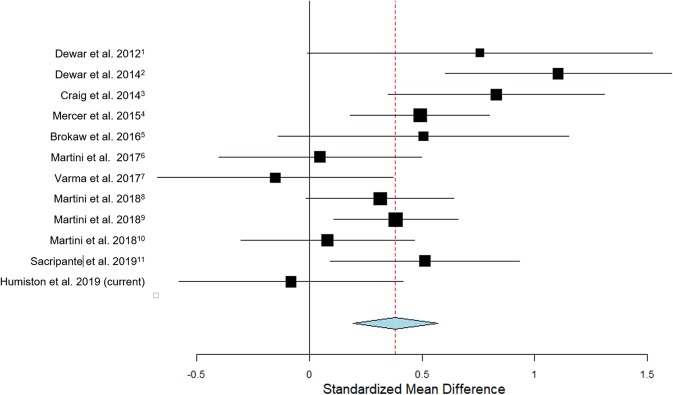
Meta-analytic summary effect. Estimated effect size +/− 95% C.I. for waking rest’s impact on verbal memory (blue diamond), calculated from the current and previous studies (black squares). Size of black square for each study is proportional to sample size, error bars represent the 95% confidence interval. Red dotted line represents point estimate for the summary effect size (0.38). For each study, the standardized mean difference was calculated as: $\frac{Mea{n}_{REST}-Mea{n}_{CONTROL}}{S{D}_{POOLED}}$.

To assess the degree to which our current observations are consistent with previous findings, Fig. 4 plots the current effect alongside those of 11 previous studies that also examined the effect of a brief period of waking rest on memory in healthy control participants (see Methods). Using a continuous random-effects model, the summary effect is significantly greater than zero (d = 0.38, p = 0.001; see Supplementary Table S8), and falls within the 95% confidence interval of the current study’s effect.

## Discussion

Contrary to expectations, we were not able to detect a memory benefit of waking rest in the present study. This contradicts our own previously-reported finding^1^, and the results of several other recent studies^1–4,8,9^, in which waking rest led to an improvement in memory. However, it is in line with a handful of other studies in which the benefit of rest for memory was absent or conditional on repeated testing^13,35,36^.

Failure to detect a significant effect in the current study could have been the simple result of sampling and/or measurement error, combined with inadequate power to detect a smaller-than-expected effect. Figure 4 illustrates that, while not statistically significant, the currently observed effect does lie within the 95% confidence interval of that reported by Brokaw *et al*.^1^. Thus, our current effect size is within the range of what would be expected based on this prior effect.

The summary effect computed from all available published studies is statistically significant, but suggests that the size of the effect of rest on verbal memory may be smaller than previously thought (d = 0.38 for the current summary effect, as opposed to d = 0.51 for Brokaw *et al*.^1^). This may provide a more accurate estimate of the true size of rest’s effect than any single previous study. Taken together, the aggregated evidence is consistent with the existence of a memory benefit of waking rest. Given that effect sizes are often overestimated in early published studies describing a new effect of interest^43^, it is not surprising for subsequent research to reduce the estimate. Knowledge that the true effect of post-training rest on verbal memory may be in the medium-to-small effect range can usefully inform the planning of future studies, enabling researchers to plan for sample sizes that will power studies sufficiently to detect this effect.

Some of the observed variability in effect size across could also be driven by heterogeneity within the relatively small group of studies included in the meta-analysis, including inter-study variability in participant age, testing procedures, and the particular type of memory task utilized (see Table S10). However, the extent to which any of these factors mediate rest’s effect on memory remains unknown.

In contrast to Brokaw *et al*.^1^, we also failed to detect an association between memory and degree of focus on Snood during the distractor condition, which may not be surprising given our failure to detect an overall effect of rest on memory in this sample. However, we also report the novel observation that participants who report *less frequent* daydreaming show *improved* memory across the rest interval. This is of interest in light of the established association between daydream frequency and individual differences in “default network” activation during rest^44,45^. The default network, a set of brain regions that tend to be active in the absence of directed cognitive tasks, has been associated with the generation of spontaneous thought and imagery^46,47^. We and others have hypothesized that activation of this network, and the associated generation of spontaneous thought and imagery, might positively contribute to memory consolidation^38,45,48^. Yet our current observation suggests just the opposite – that increased daydreaming and spontaneous thought processes resulted in *impaired* consolidation across a resting interval.

At first glance, this would seem to argue against a memory benefit of rest, as periods of rest clearly allow for more daydreaming than periods of active wake. However, it may be that waking rest does benefit memory, but selectively during periods when the mind is “blank” (an absence of spontaneous thought), a state that was negatively associated with daydream frequency in the current study.

The hippocampus is a crucial element of the default network, and its engagement has been hypothesized to inhibit offline consolidation from occurring^7^. Accordingly, it is possible that hippocampal resource engagement during daydreaming and mindwandering functions to interfere with memory consolidation. This hypothesis is in line with two recent studies showing that engaging the hippocampal memory system via an autobiographical memory recall task impairs memory for previously learned information, even in the absence of directed attention to any external task^14,15^. Thus, it may be that the particular state of resting wakefulness that most strongly benefits memory is one during which spontaneous cognition is at a minimum and the default network is not strongly engaged.

In summary, together with previous studies, our current observations help to more accurately estimate the effect of waking rest on memory. Although large effect sizes were reported in early studies with this same experimental design, subsequent work, including the present failed replication, moves us toward a more modestly sized estimate of rest’s impact on verbal memory. This summary effect (d = 0.38) can serve as a useful guide for planning sufficiently powered future studies.

## Methods

All study procedures and analysis methods described here were preregistered on Open Science Framework prior to beginning data collection. The preregistration is publicly available here: https://osf.io/w7x4p/.

### Participants and sample size

A target sample size of n = 32 participants was set by determining the number of participants needed to achieve power of 0.90 for detecting the within-subjects effect of post-training rest vs. distractor task on raw improvement in memory score reported in Brokaw *et al*.^1^ (*d*_*z*_ = 0.60).

Participants (n = 48) were tested in small group sessions of up to 5. Enrollment continued until we reached the target number of participants meeting our preregistered inclusion criteria of: Correctly following study instructions (n = 4 enrolled participants excluded), obtaining an average ≥5 hours of sleep on the 3 nights preceding participation (n = 1 enrolled participants excluded, calculated from self-reported data in a 3-day retrospective sleep log), scoring ≤5 on the baseline Stanford Sleepiness Scale^49^ (SSS, n = 3 enrolled participants excluded), speaking English as their native language (n = 2 enrolled participants excluded); and having not already participated in a study using the same story task (n = 2 enrolled participants excluded). We concluded enrollment when we had reached n = 35 usable participants according to these criteria.

An additional n = 4 participants had to be excluded for outlying raw improvement scores (>1.5 quartiles from the median, also preregistered). Thus, we arrived at a final sample size of n = 31 participants (22 females, mean age of 18.90 ± 1.22 SD, range 18–22), who slept an average of 8.21 ± 0.91 SD hours on the three nights preceding the experiment; see Table 2 and Supplementary Table S9).

**Table 2:** Participant characteristics.

	mean	±SD
Age (yrs)	18.9	1.2
ESS	14.7	4.0
SSS^a^	2.5	0.7
MAAS	3.8	0.6
DFS	35.2	9.8
Sex (% male)	29%	

^a^Stanford Sleepiness Scale at baseline. ESS = Epworth Sleepiness Scale; MAAS = Mindfulness Attention Awareness Scale^40^, DFS = Daydreaming Frequency Scale^41^.

Participants were recruited by email, advertisement, or word-of-mouth, and were compensated by receiving either $10/hr or credit for an introductory psychology course. All participants signed written informed consent. The study was approved by the Furman University Institutional Review Board, and the methods were carried out in accordance with the relevant guidelines and regulations.

### Procedure

**Figure 5:**
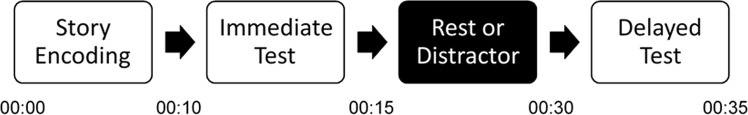
Study Timeline. Times represent minutes elapsed since the start of the study. Participants listened to a short story prior to a 15 min retention interval, during which they either rested with their eyes closed or completed a distractor task. Participants were tested on their recall of the story immediately before and after the retention interval. Times represent minutes elapsed since the start of the study.

The experimental timeline is illustrated in Fig. 5. The study was a preregistered within-subjects design, with all participants completing the rest condition and the distractor condition. Participants completed each condition on a separate laboratory visit (order of conditions counterbalanced; 55% did the rest condition first). On each visit, participants were exposed to a different version of the story task (Story A or Story B), and assignment of story version to experimental condition was also counterbalanced (55% did Story A first). Visits were separated by at least 24 hrs (average number of days between visits = 1.16 ± 0.74 SD). All study tasks and forms, with the exception of the distractor task, were completed using Qualtrics survey software^50^. Participants were tested in group sessions of up to 5 individuals in a computer-lab setting. All participants within a single session were assigned to the same condition and counterbalancing order (e.g., waking rest and Story A first, and distractor task and Story B second). There were four possible counterbalancing orders – waking rest or distractor task first and Story A or Story B first – which were assigned to experimental sessions in a block randomized order. Counterbalancing order had no effect on immediate or delayed story recall performance in either condition.

The procedure followed that of Brokaw *et al*.^1^ closely, differing only in that (1) the current study did not include EEG recording, (2) the instructions for the distractor task were given at the beginning of both sessions, (3) up to five participants could complete the study together, and (4) there was a minimum 24 hr delay between conditions.

Upon arrival at the laboratory, participants signed informed consent, then completed initial paperwork including a demographic survey, the Epworth Sleepiness Scale (ESS; a measure of trait sleepiness)^51^, and a 3-day retrospective sleep log. The experimenter then explained the rules of the distractor task, the computer game “Snood” (www.snoodworld.com). Participants practiced the game for one minute, then completed the Stanford Sleepiness Scale (SSS), a measure of state sleepiness.

The experimenter then played an audio file of a short story (approx. 30 sec) about a school cafeteria worker who was robbed (Story A) or a truck driver who skidded off the road (Story B)^42^. Immediately afterward, participants completed an initial free recall test (immediate test) during which they attempted to recall as many of the details of the story as possible. Afterward, participants began the 15 min retention interval, during which they either rested with their eyes closed or completed the distractor task (Snood). In the rest condition, participants were instructed to sit upright in their chairs with their eyes closed and to remain awake. They were not given any instructions about what to do mentally during the interval. In the distractor condition, participants played Snood for 15 min, starting a new game every time they won or lost. Immediately following the experimental phase, participants were given a delayed recall test. The research assistant was present in the room with participants for the duration of the experiment, and monitored participants to ensure they were complying with instructions (e.g. remaining sitting upright with eyes closed in the rest condition and playing Snood for the entirety of the distractor task condition).

Following the delayed recall test, participants completed the post-test SSS and completed an exit questionnaire that assessed subjective experience during the retention interval (Supplementary Materials). This questionnaire included multiple-choice questions about whether participants rehearsed the story and followed task instructions, about their “thoughts, feelings, or daydreams” during the interval, and the proportion of the interval they spent in one or more of 13 pre-defined mental categories: “thinking about the past” (something else earlier today/yesterday to a week ago/past year or several years ago), “imagining the future” (later today/tomorrow to next week/next year or several years), “thinking about the short story”, “thinking about [playing Snood]/[resting with eyes closed]”, “counting the time”, “mind was blank”, “meditating”, “sleeping”, “thinking about something else”, and “other”.

The first session ended after participants completed the exit questionnaire. Following the second session, participants also completed the Mindfulness Attention Awareness Scale (MAAS)^40^ and the Daydreaming Subscale of the Imaginal Processes Inventory (DFS)^41^. They were then debriefed and compensated.

Detailed information about the short story task and Snood can be found in Supplementary Materials.

#### Calculation of meta-analytic summary effect

To integrate our current findings with the prior literature, we additionally calculated a meta-analytic summary effect across all studies of the impact of waking rest on verbal memory that used the same fundamental experimental design. This analysis was not pre-registered. Based on a literature search (using the Academic Search Premier, PsychARTICLES, Psychology and Behavioural Sciences Collection, PsychINFO, and Science Reference Center databases), we identified n = 11 prior studies that also tested the effect of a short (5–20 min) period of post-learning eyes closed rest on verbal memory in healthy controls, as compared to a visuomotor task control condition.

Details of the included studies are described in Supplementary Table S10. For each study, we considered only the rest and control conditions best matching those used in our own design, omitting other comparison conditions that may have been present. For each study, the effect included for meta-analysis was the difference between memory change across rest and memory change across the control condition. If multiple measures of memory were tested, we selected either *1)* the measure described as the primary outcome, or if that was not clear, *2)* the first measure reported. If memory was tested at multiple time points (e.g. both immediately after rest and a week later), we selected the time point most comparable to that used in the current study (i.e., immediately after rest). If multiple age groups were tested, we selected the effect that encompassed all ages tested, or if that information was not available, we selected the effect for the age group most comparable to that of our current study (young adults). All effect sizes were converted to standardized mean differences (SMD: $\frac{Mea{n}_{REST}-Mea{n}_{CONTROL}}{S{D}_{POOLED}}$), and a continuous random effects model was used to compute a summary effect using OpenMeta Analyst: www.cebm.brown.edu/openmeta.

## Supplementary information

Supplementary Information

## Data Availability

The datasets generated during and/or analysed during the current study are available from the Open Science Framework repository, https://osf.io/p2gw6/.
