# Supplementary material for: Resting States and Memory Consolidation: A Preregistered Replication and Meta-Analysis
Source: Sci Rep. 2019 Dec 18;9:19345. doi: 10.1038/s41598-019-56033-6 (PMC6920145; doi:10.1038/s41598-019-56033-6)
Supplement: Supplementary file 1 — Supplementary Information [file 41598_2019_56033_MOESM1_ESM.docx]

**Resting States and Memory Consolidation:**

**A Preregistered Replication and Meta-Analysis**

**Supplementary Text and Tables**

Graelyn B. Humiston^1^, Matthew A. Tucker^2^, Theodore Summer^1^, and Erin J. Wamsley^1*^

^1^Department of Psychology and Program in Neuroscience, Furman University, Greenville, South Carolina, United States

^2^Department of Biomedical Sciences, University of South Carolina School of Medicine, Greenville, South Carolina, United States

* Corresponding author

E-mail: [erin.wamsley@furman.edu](mailto:erin.wamsley@furman.edu) (EW)

Contents

[Preregistered* exploratory analyses 2](#_Toc13743814)

[Additional text and tables 10](#_Toc13743815)

[Story recall task supplementary methods 10](#_Toc13743816)

[Snood supplementary methods 11](#_Toc13743817)

[References 13](#_Toc13743818)

[Exit Questionnaire 14](#_Toc13743819)

# Preregistered* exploratory analyses

**Except where otherwise noted*

**Table S1.** Supplementary story recall measures.

|  |  | **Waking rest** | | **Distractor** | |  | | |
| --- | --- | --- | --- | --- | --- | --- | --- | --- |
|  |  | *mean* | *±SEM* | *mean* | *±SEM* | *t* | *df* | *p* |
| **% change** | Correct recall | -4% | 1% | -3% | 2% | .27 | 30 | *.79* |
|  | False recall | -21% | 11% | -11% | 9% | .67 | 25 | *.51* |
|  | Total recall | -5% | 1% | -4% | 2% | .23 | 30 | *.82* |
| **Raw change** | False recall | -.02 | .17 | -.08 | .14 | .28 | 30 | *.78* |
| **Baseline** | False recall | 1.73 | .19 | 2.04 | .25 | 1.29 | 30 | *.21* |

*Notes.* Paired-samples *t*-tests comparing change in correct, false, and total recall over the 15min interval, as well as baseline false recall, between the waking rest and distractor task conditions. Baseline false recall is the number of details falsely recalled on the initial free recall test.

**Table S2.** Non-preregistered correlation of trait daydreaming with subjective experience during waking rest.

|  | *r(31)* | *p* |
| --- | --- | --- |
| The story from earlier | -.04 | *.82* |
| Something that happened earlier today | .03 | *.89* |
| Something that happened yesterday to a week ago | -.11 | *.55* |
| Something that happened last year or several years ago | .08 | *.68* |
| The remainder of the day | .04 | *.84* |
| Something that will happen tomorrow to next week | .01 | *.95* |
| Something that will happen in the next year or several years | .11 | *.56* |
| Resting with eyes closed | -.22 | *.23* |
| My mind was blank | -.42 | *.02** |
| I was counting the time | .07 | *.71* |
| I was doing focused meditation | .23 | *.21* |
| I was sleeping | .28 | *.13* |
| Other | .30 | *.10* |

*Notes.* Participants reported the proportion of time spent in 13 pre-defined mental categories in an exit questionnaire at the end of each session. *P-*values are based on Pearson’s correlations between the Daydreaming Frequency Scale (DFS) and the proportion of time spent thinking about each of the items on the exit questionnaire in the waking rest condition. **p*<.05

**Table S3.** Correlation of mindfulness and trait daydreaming with recall measures.

|  |  |  | **Mindfulness Attention Awareness Scale** | |  | **Daydreaming Frequency Scale** | |
| --- | --- | --- | --- | --- | --- | --- | --- |
|  |  | *N* | *r* | *p* |  | *r* | *p* |
|  | Daydreaming Frequency Scale | 31 | -.48 | *.006*** |  |  |  |
| **Correct recall** | Raw change over waking rest | 31 | .31 | *.09* |  | -.51 | *.004*** |
|  | % change over waking rest | 31 | .19 | *.31* |  | -.47 | *.008*** |
|  | Raw change over the distractor task | 31 | .26 | *.15* |  | -.26 | *.16* |
|  | % change over the distractor task | 31 | .16 | *.39* |  | -.21 | *.26* |
| **False recall** | Raw change over waking rest | 31 | -.17 | *.36* |  | .28 | *.13* |
|  | % change over waking rest | 27 | -.39 | *.045** |  | .41 | *.03** |
|  | Raw change over the distractor task | 31 | -.11 | *.55* |  | -.25 | *.17* |
|  | % change over the distractor task | 28 | -.12 | *.54* |  | -.17 | *.39* |
| **Total recall** | Raw change over waking rest | 31 | .12 | *.52* |  | -.19 | *.31* |
|  | % change over waking rest | 31 | .04 | *.83* |  | -.02 | *.90* |
|  | Raw change over the distractor task | 31 | .16 | *.39* |  | -.39 | *.03** |
|  | % change over the distractor task | 31 | .08 | *.67* |  | -.30 | *.10* |

*Notes.* Pearson’s correlations of Mindfulness Attention Awareness Scale, Daydreaming Frequency Scale, and raw and % change in correct, false, and total recall in the waking rest and distractor task conditions. **p*<.05, ***p*<.01

**Table S4.** Self-reported rehearsal.

|  | **Waking rest** | | **Distractor** | |  |  |  |
| --- | --- | --- | --- | --- | --- | --- | --- |
| **Questionnaire item** | *mean* | *±SEM* | *mean* | *±SEM* | *t* | *df* | *p* |
| Think about the story | 2.03 | .16 | 1.52 | .14 | 2.99 | *30* | *.006*** |
| Imagine the story | 1.77 | .15 | 1.35 | .10 | 2.89 | *30* | *.007*** |
| Try to remember the story | 2.00 | .17 | 1.68 | .17 | 1.90 | *30* | *.07* |

*Notes.* Participants reported whether they rehearsed – thought about, imagined, or tried to remember – the story on the rehearsal questionnaire. *P-*values are based on paired-samples *t*-tests comparing answers to the rehearsal questionnaire between the waking rest and distractor conditions. ***p*<.01

**Table S5.** Comparison of subjective experience.

|  | **Waking rest** | |  | **Distractor** | |  |  | |
| --- | --- | --- | --- | --- | --- | --- | --- | --- |
| **Post-learning subjective experience item** | *mean* | *±SEM* |  | *mean* | *±SEM* |  | *t(30)* | *p* |
| The story from earlier | 4.74 | 1.43 |  | 4.39 | 1.90 |  | .18 | *.86* |
| Thinking about the past^1^ | 20.48 | 3.79 |  | 10.48 | 3.14 |  | 2.56 | *.02** |
| Something that happened earlier today | 8.90 | 2.31 |  | 7.10 | 2.38 |  | .66 | *.58* |
| Something that happened yesterday to a week ago | 9.03 | 2.65 |  | 2.90 | 1.75 |  | 1.97 | *.06* |
| Something that happened last year or several years ago | 2.55 | 1.12 |  | .48 | .48 |  | 2.36 | *.03** |
| Thinking about the future^2^ | 37.00 | 4.64 |  | 17.58 | 4.63 |  | 3.10 | *.004*** |
| The remainder of the day | 24.35 | 3.51 |  | 12.81 | 3.25 |  | 2.48 | *.02** |
| Something that will happen tomorrow to next week | 11.03 | 2.42 |  | 4.77 | 1.63 |  | 2.42 | *.02** |
| Something that will happen in the next year or several years | 1.61 | .85 |  | .00 | .00 |  | 1.90 | *.07* |
| The current task (“playing Snood” or “resting with eyes closed”) | 12.45 | 3.06 |  | 56.68 | 6.89 |  | 6.38 | *<.001**** |
| My mind was blank | 6.71 | 3.33 |  | 3.87 | 3.24 |  | .60 | *.56* |
| I was counting the time | 5.00 | 1.40 |  | 1.03 | .71 |  | 2.42 | *.02** |
| I was doing focused meditation | 3.55 | 1.56 |  | .00 | .00 |  | 2.27 | *.03** |
| I was sleeping | 1.94 | 1.94 |  | .48 | .48 |  | .72 | *.48* |
| Other | 8.13 | 3.46 |  | 5.48 | 3.59 |  | .60 | *.56* |

*Notes.* Participants reported the proportion of time spent in 13 pre-defined mental categories in an exit questionnaire at the end of each session. *P*-values are based on paired-samples *t*-tests comparing the waking rest and distractor task conditions. ^1^Average time spent thinking about “Something that happened earlier today”, “Something that happened yesterday to a week ago”, and “Something that happened last year or several years ago”. ^2^Average time spent thinking about “The remainder of the day”, “Something that will happen tomorrow to next week”, and “Something that will happen in the next year or several years”. **p*<.05, ***p*<.01, ****p*<.001

**Table S6.** Correlations of subjective experience and change in recall during the waking rest and distractor conditions.

|  | **Waking rest** | |  | **Distractor** | | |
| --- | --- | --- | --- | --- | --- | --- |
| **Post-learning subjective experience item** | *r(31)* | *p* |  | *r(31)* | *p* |  |
| The story from earlier | .03 | *.87* |  | -.21 | *.26* |  |
| Thinking about the past^1^ | .04 | *.82* |  | .02 | *.90* |  |
| Something that happened earlier today | -.08 | *.68* |  | -.01 | *.95* |  |
| Something that happened yesterday to a week ago | .10 | *.60* |  | .06 | *.75* |  |
| Something that happened last year or several years ago | .07 | *.72* |  | -.01 | *.97* |  |
| Thinking about the future^2^ | -.22 | *.23* |  | .14 | *.44* |  |
| The remainder of the day | -.18 | *.33* |  | .04 | *.84* |  |
| Something that will happen tomorrow to next week | -.05 | *.79* |  | .33 | *.07* |  |
| Something that will happen in the next year or several years^3^ | -.33 | *.07* |  | . | *.* |  |
| The current task (“playing Snood” or “resting with eyes closed”)^4^ | .11 | *.55* |  | -.07 | *.70* |  |
| My mind was blank | .39 | *.03* |  | .01 | *.95* |  |
| I was counting the time | -.04 | *.82* |  | -.34 | *.06* |  |
| I was doing focused meditation | .12 | *.53* |  | . | *.* |  |
| I was sleeping | -.42 | *.02** |  | -.01 | *.97* |  |
| Other | -.04 | *.84* |  | .10 | *.59* |  |

*Notes.* Participants (n=31) reported the proportion of time spent in 15 pre-defined mental categories in an exit questionnaire at the end of each session (the preregistration erroneously stated that there were 16 categories). Pearson’s correlations assessed whether raw change in correct recall over the waking rest or the distractor condition was associated with each category in the corresponding exit questionnaire. ^1^Average time spent thinking about “Something that happened earlier today”, “Something that happened yesterday to a week ago”, and “Something that happened last year or several years ago”. ^2^Average time spent thinking about “The remainder of the day”, “Something that will happen tomorrow to next week”, and “Something that will happen in the next year or several years. Blank cells indicate that no participant endorsed this thought category in the distractor condition. **p*<.05

**Table S7.** Story rehearsal and change in recall.

|  |  |  | **Thought about story** | | |  | **Did not think about story** | | |  |  |  |  |
| --- | --- | --- | --- | --- | --- | --- | --- | --- | --- | --- | --- | --- | --- |
|  |  |  | *N* | *mean* | *±SD* |  | *N* | *mean* | *±SD* |  | *t* | *df* | *p* |
| **Distractor task** | *Raw change* |  |  |  |  |  |  |  |  |  |  |  |  |
|  |  | Correct | 12 | -.79 | 1.10 |  | 19 | -.26 | .87 |  | 1.49 | 29 | *.15* |
|  |  | False | 12 | .17 | .58 |  | 19 | -.24 | .73 |  | 1.6 | 29 | *.12* |
|  |  | Total | 12 | -.63 | 1.32 |  | 19 | -.50 | 1.01 |  | .30 | 29 | *.77* |
|  | *% change* |  |  |  |  |  |  |  |  |  |  |  |  |
|  |  | Correct | 12 | -6.1% | 8.2% |  | 19 | -1.5% | 8.6% |  | 1.47 | 29 | *.15* |
|  |  | False | 10 | 8.2% | 32% |  | 18 | -21% | 45% |  | 1.83 | 26 | *.08* |
|  |  | Total | 12 | -5.4% | 9.9% |  | 19 | -3.3% | 7.6% |  | .67 | 29 | *.51* |
| **Waking rest** | *Raw change* |  |  |  |  |  |  |  |  |  |  |  |  |
|  |  | Correct | 21 | -.43 | .91 |  | 10 | -.80 | .75 |  | 1.12 | 29 | *.27* |
|  |  | False | 21 | -.10 | .82 |  | 10 | .15 | 1.16 |  | .68 | 29 | *.50* |
|  |  | Total | 21 | -.52 | .87 |  | 10 | -.65 | 1.13 |  | .34 | 29 | *.74* |
|  | *% change* |  |  |  |  |  |  |  |  |  |  |  |  |
|  |  | Correct | 21 | -2.5% | 6.1% |  | 10 | -6.7% | 6.8% |  | 1.73 | 29 | *.09* |
|  |  | False | 17 | -19% | 54% |  | 10 | -25% | 64% |  | .26 | 25 | *.80* |
|  |  | Total | 21 | -4.1% | 6.5% |  | 10 | -5.7% | 11% |  | .53 | 29 | *.60* |

*Notes.* Participants reported whether they thought about the story during the retention interval on the rehearsal questionnaire. *P*-values are based on paired-samples *t*-tests comparing raw and % correct, false, and total change in recall over each condition between participants who reported thinking or not thinking about the story on the rehearsal questionnaire.

**Table S8.** Effect sizes and 95% C.I.s. for all studies included in the meta-analysis.

| **Studies** | *Standardized Mean Difference* | *(95% C.I.)* |
| --- | --- | --- |
| Dewar et al. 2012^1^ | .756 | (-.010, 1.523) |
| Dewar et al. 2014^2^ | 1.105 | (.602, 1.608) |
| Craig et al. 2014^3^ | .829 | (.348, 1.310) |
| Mercer et al. 2015^4^ | .491 | (.182, 0.800) |
| Brokaw et al. 2016^5^ | .507 | (-.140, 1.153) |
| Martini et al. 2017^6^ | -.152 | (-.677, .372) |
| Varma et al. 2017^7^ | .047 | (-.403, .497) |
| Martini et al. 2018^8^ | .313 | (-.015, .642) |
| Martini et al. 2018^9^ | .383 | (.106, .660) |
| Martini et al. 2018^10^ | .080 | (-.304, .465) |
| Sacripante et al. 2019^11^ | .512 | (.092, .931) |
| Humiston et al. 2019 (current) | -.083 | (-.581, .415) |
| **Overall** | **.325** | **(.118, .532)** |

*Notes.* Estimated effect size (standardized mean difference) and 95% confidence interval for each of the studies included in the meta-analysis, as well as for the calculated summary effect.

# Additional text and tables

**Table S9.** Average sleep obtained before the study.

|  | *N* | *mean* | *±SD* |
| --- | --- | --- | --- |
| **Bed time** | 31 | 12:19am | 1:06hrs |
| **Wake time** | 31 | 8:41am | 1:06hrs |
| **Total sleep** | 31 | 8:13hrs | 0:55hrs |

*Notes.* Calculated from self-reported data in the 3-day retrospective sleep log, which was filled out during the first session.

## Story recall task supplementary methods

The story recall task was identical to that used in Brokaw et al.^5^ and Dewar et al^1^, which was adapted from the Wechsler Memory Scale^12^. Two short story versions (A and B) were used, one for each condition, with assignment to condition counterbalanced across participants. Neither immediate nor delayed recall scores differed significantly between story versions (p>.12).

Participants listened to a short story (30sec) played from a speaker connected to a laptop in the testing room, with the volume set to be clearly audible to everyone in the room. Immediately after the story was finished, participants were tested on recall, with the instructions: “In the textbox, type everything you can remember about the story that you just heard, starting at the beginning. Please take your time and include all of the details you can remember. Type everything you can remember, even if you are not sure that it is correct. You have 5 minutes to type your response, please try to use all of this time. If you finish early, click the next button and sit quietly”. Though the instructions specified a 5min time limit, participants were given more time if they were still typing after 5min had elapsed. Participants were given a delayed recall test, following the same format, immediately after the retention interval. For the delayed recall test, the experimenter emphasized that participants were to again write down every detail they could remember. The time limit was again stated to be 5min, but more time was given if needed.

## Snood supplementary methods

The object of the computer game “Snood” ([www.snoodworld.com](http://www.snoodworld.com)) is to group three or more of the same icons (snoods) together in order to clear them from the screen. The difficulty level was set to medium, and participants started a new game at the same difficulty level each time they won or lost within the 15min retention interval. Snood was used as the distractor in the current study and in Brokaw et al.^5^ because it is a visuospatial task with minimal motor components, and has no content overlap with the short story task. It is also engaging without imposing a large cognitive load, minimizing the possibility of interference with the short story task.

**Table S10.** Summary of studies included in meta-analysis.

| **Citation** | **Procedures summary** | **Design** | **N** | **Effect selected for effect-size calculation** |
| --- | --- | --- | --- | --- |
| Dewar et al. (2012)^1^ | (Experiment 1) Older adults (mean 73yrs) listened to a short story, then rested wakefully or completed a spot-the-difference task for 10min. | Within | 14 | Mean change in recall score across waking rest condition vs spot-the difference condition, at the 15-30min recall test. |
| Dewar et al. (2014)^2^ | (Experiment 1) Older adults (mean 73yrs) learned a list of 15 nouns, then rested wakefully or completed a spot-the-difference task for 10min. | Between | 70 | Mean words recalled in the rest vs spot-the-difference groups, at the 15min recall test. |
| Craig et al. (2014)^3^ | (Experiment 1) Young adults (mean 22yrs) learned lists of 15 nouns, and then either rested wakefully or completed a picture search task for 9mins. | Within | 36 | % of words retained at delayed recall in the rest condition vs the picture search condition (~9-27min delay). |
| Mercer (2015)^4^ | Adults (mean 21yrs) learned a set of English-Icelandic word pairs, then rested wakefully for 8min either before or after completing a similar or dissimilar distractor task. | Within | 83 | Mean forgetting in pre-interference vs post-interference rest condition, at the ~19min delayed recall test. |
| Brokaw et al. (2016)^5^ | Young adults (mean 20yrs) listened to a short story, then rested wakefully or played a simple computer game for 15min. | Within | 19 | Mean change in recall scores across waking rest vs distractor task conditions, at the 15min recall test. |
| Martini et al. (2017)^6^ | (Experiment 1) Young adults (mean 23yrs) read a short story, then rested wakefully or performed a visual distractor task for 8min. | Within | 28 | Mean change in free recall of rest vs distractor conditions at the 7-day recall test. |
| Varma et al. (2017)^7^ | (Experiment 1) Young adults (mean 22yrs) learned 90 object-word pairs, then rested wakefully or completed a 2-back task for 12min. | Within | 38 | Mean d-prime memory scores of unfilled and filled delay conditions at the ~40-50min recognition test. |
| Martini et al. (2018)^8^ | Young (mean 21yrs) and older (mean 69yrs) adults learned a list of 15 nouns, then rested wakefully or solved matrices for 8min. | Within | 72 | Mean words recalled in the rest vs distractor conditions at the 15-30min recall test. |
| Martini et al. (2018)^9^ | Children (age 13-14) learned a list of 15 nouns, then rested wakefully or solved abstract visuo-spatial problems for 10min. | Within | 102 | Mean words recalled in the rest vs problem-solving conditions at the 7-day recall test. |
| Martini et al. (2018)^10^ | (Experiment 1) Young adults (mean 22yrs) learned a list of 15 nouns, then rested wakefully or solved matrices for 8min. | Within | 52 | Mean words recalled in the rest vs problem solving conditions at the ~21min delayed recall test. |
| Sacripante et al. (2019)^11^ | Young adults and teens (mean 17yrs) listened to 3 audio stories, and then either completed a spot-the-difference task for 10min, or rested wakefully for 9min followed by 1min of the spot-the-difference task. | Within | 45 | Mean “gist” elements recalled during the 10min delayed verbal recall test. |

# References

1. Dewar, M., Alber, J., Butler, C., Cowan, N. & Della Sala, S. Brief wakeful resting boosts new memories over the long term. *Psychological Science* **23**, 955–960 (2012).

2. Dewar, M., Alber, J., Cowan, N. & Della Sala, S. Boosting long-term memory via wakeful rest: Intentional rehearsal is not necessary, consolidation is sufficient. *PLoS ONE* **9**, 1–10 (2014).

3. Craig, M., Della Sala, S. & Dewar, M. Autobiographical thinking interferes with episodic memory consolidation. *PLoS ONE* **9**, e93915 (2014).

4. Mercer, T. Wakeful rest alleviates interference-based forgetting. *Memory* **23**, 127–137 (2015).

5. Brokaw, K. *et al.* Resting state EEG correlates of memory consolidation. *Neurobiology of Learning and Memory* **130**, 17–25 (2016).

6. Martini, M., Riedlsperger, B., Maran, T. & Sachse, P. The effect of post-learning wakeful rest on the retention of second language learning material over the long term. *Curr Psychol* (2017). doi:10.1007/s12144-017-9760-z

7. Varma, S. *et al.* Non-interfering effects of active post-encoding tasks on episodic memory consolidation in humans. *Front. Behav. Neurosci.* **11**, (2017).

8. Martini, M., Zamarian, L., Sachse, P., Martini, C. & Delazer, M. Wakeful resting and memory retention: A study with healthy older and younger adults. *Cognitive Processing* (2018). doi:10.1007/s10339-018-0891-4

9. Martini, M., Martini, C., Bernegger, C. & Sachse, P. Post‐encoding wakeful resting supports the retention of new verbal memories in children aged 13–14 years. *British Journal of Developmental Psychology* (2018). doi:10.1111/bjdp.12267

10. Martini, M., Martini, C., Maran, T. & Sachse, P. Effects of post-encoding wakeful rest and study time on long-term memory performance. *Journal of Cognitive Psychology* **30**, 558–569 (2018).

11. Sacripante, R., McIntosh, R. D. & Della Sala, S. Benefit of wakeful resting on gist and peripheral memory retrieval in healthy younger and older adults. *Neuroscience Letters* **705**, 27–32 (2019).

12. Wechsler, D. *WMS-R: Wechsler Memory Scale-Revised: Manual*. (Harcourt Brace Jovanovich, 1987).

# Exit Questionnaire

***This questionnaire, along with the rest of the study, was completed in Qualtrics. [**italicized text] indicates notes that did not appear in the Qualtrics questionnaire.*

*****Rehearsal questionnaire***

In between when you first saw the picture pairs, and when you were tested on them again, how often did you think about picture pairs?

- Not at all
- Once or twice
- A few times
- A lot
- Constantly

***Displayed only if “Not at all” is NOT selected*

When you thought about the picture pairs, would you consider these thoughts:

- Spontaneous (you weren't really trying to think about it, but the thoughts just popped into mind)
- Intentional (you were intentionally trying to think about it)
- Both

In between when you first saw the picture pairs, and when you were tested on them again, how often did you imagine the picture pairs?

- Not at all
- Once or twice
- A few times
- A lot
- Constantly

***Displayed only if “Not at all” is NOT selected*

When you imagined the picture pairs, would you consider these thoughts:

- Spontaneous (you weren't really trying to imagine it, but the image just popped into mind)
- Intentional (you were intentionally trying to imagine it)
- Both

In between when you first saw the picture pairs, and when you were tested on them again, how often did you try to remember the picture pairs?

- Not at all
- Once or twice
- A few times
- A lot
- Constantly

***Displayed only if “Not at all” is NOT selected*

When you tried to remember the word pairs, would you consider these thoughts:

- Spontaneous (you weren't really trying to remember it, but the thought just popped into mind)
- Intentional (you were intentionally trying to remember it)
- Both

*****Post-learning subjective experience***

Please answer honestly—your answer to this question does not affect your credit or payment for this study:

Which of the following were you doing during the break period? (Select all that apply)

- Thinking about the story from earlier
- Thinking about something that happened earlier today
- Thinking about something that happened yesterday to a week ago
- Thinking about something that happened last year or several years ago
- Thinking about the remainder of the day
- Thinking about something that will happen tomorrow to next week
- Thinking about something that will happen in the next year or several years
- Thinking about [playing Snood/resting with eyes closed]
- My mind was blank
- I was counting the time
- I was doing focused meditation
- I was sleeping
- Other ____________________

What proportion of the time did you spend thinking about each item? (Total must be 100)

***Responses to each item were given on a slider scale, and proportions were computed automatically in Qualtrics*

______ Thinking about the story from earlier

______ Thinking about something that happened earlier today

______ Thinking about something that happened yesterday to a week ago

______ Thinking about something that happened last year or several years ago

______ Thinking about the remainder of the day

______ Thinking about something that will happen tomorrow to next week

______ Thinking about something that will happen in the next year or several years

______ Thinking about [playing Snood/resting with eyes closed]

______ My mind was blank

______ I was counting the time

______ I was doing focused meditation

______ I was sleeping

______ Other

During the break, you were asked to [insert appropriate activity here]. How much of the period did you actually spend doing this?

- The entire time
- At least 75% of the time
- At least half of the time
- At least 25% of the time
- Less than 25% of the time
- I didn't follow that instruction at all

Please describe your thoughts, feelings, or daydreams while [playing Snood/you were resting with your eyes closed] in as much detail as you can remember:

***Participants were given an open answer field with to type their response*
